# *Legionella* Pneumonia in the Molecular Diagnostics Era: From Intracellular Pathogenesis to Precision Management

**DOI:** 10.3390/diagnostics16213677

**Published:** 2026-11-06

**Authors:** Lei Liu, Bingbing Zhang, Kai Sang, Jiyong Lin, Mingshun Li, Peiling Zhong, Na Huang, Jianping Lai, Liubin Xu, Liang Chen, Yuxiang Liu, Sheng Chen

**Affiliations:** 1 Department of Infectious Diseases, Shenzhen Traditional Chinese Medicine Hospital, The Fourth Clinical Medical College of Guangzhou University of Chinese Medicine, Shenzhen 518033, China; 2 Department of Emergency, Shenzhen Traditional Chinese Medicine Hospital, The Fourth Clinical Medical College of Guangzhou University of Chinese Medicine, Shenzhen 518033, China; gsjlyx111@163.com; 3 Department of Respiratory and Critical Care Medicine, Shenzhen Traditional Chinese Medicine Hospital, The Fourth Clinical Medical College of Guangzhou University of Chinese Medicine, Shenzhen 518033, China

**Keywords:** *Legionella pneumophila*, Legionnaires’ disease, metagenomic next-generation sequencing, diagnostic stewardship, intracellular pathogenesis, precision antimicrobial management, urinary antigen

## Abstract

*Legionella pneumophila* is a major cause of severe community-acquired pneumonia and one of the most lethal respiratory pathogens. Nevertheless, it remains substantially underdiagnosed because conventional diagnostic assays are slow, insensitive, or limited to specific serogroups. In this narrative review, we integrate fragmented literature regarding *Legionella* pneumonia, covering its intracellular pathogenesis, clinical spectrum and patient management. Evidence was identified through a structured search of PubMed/MEDLINE and Europe PMC (23 September 2026) and prioritized by design, with primary clinical and mechanistic studies and systematic reviews taking precedence over narrative summaries and with society guidelines used to anchor management recommendations. We argue that this field is undergoing a diagnostic paradigm shift fueled by culture-independent molecular assays. We first review the Dot/Icm type IV secretion system and intracellular lifecycle that allow *Legionella* to replicate within macrophages while evading host innate immunity. We then summarize the full clinical spectrum, ranging from self-limited Pontiac fever to fatal Legionnaires’ disease complicated by acute respiratory distress syndrome, as well as epidemiological characteristics including waterborne transmission and outbreak risks. The core of this review examines how metagenomic next-generation sequencing, targeted NGS, plasma microbial cell-free DNA and syndromic multiplex PCR are reshaping diagnostic workflows, and compares the strengths and limitations of these novel tools with urinary antigen testing and bacterial culture. Pooled estimates place the sensitivity of urinary antigen testing at 0.79 overall and 0.86 for serogroup 1, and that of respiratory PCR at 97.4%, with specificities of 1.00 and 0.99, respectively. We further evaluate contemporary therapeutic evidence, including fluoroquinolone versus macrolide monotherapy, combination regimens, and adjunctive corticosteroid therapy. Throughout this review, we highlight that *Legionella* is a pathogen whose clinical presentation, diagnostic yield and prognosis are highly dependent on host immune status, exposure setting and appropriate diagnostic stewardship. We conclude by identifying critical knowledge gaps and outlining a framework for diagnosis-driven precision antimicrobial management of this underrecognized pathogen. That framework is an evidence-informed synthesis rather than a validated pathway: no prospective study has yet shown that it improves patient outcomes, and its cost-effectiveness has not been measured.

## 1. Introduction

*Legionella pneumophila* occupies a unique position among respiratory pathogens. Human-to-human transmission is extremely rare and not considered a major transmission route; it does not colonize the upper airway and causes disease almost exclusively through the inhalation of contaminated water aerosols. Nevertheless, it contributes a disproportionate burden of severe community-acquired pneumonia (CAP) requiring intensive care admission [1]. First identified following the 1976 American Legion convention outbreak in Philadelphia [2], Legionnaires’ disease has since been recognized worldwide as both sporadic and an outbreak-associated cause of pneumonia after the isolation and characterization of the causative bacterium [3]. Despite decades of research and the availability of targeted therapy, *Legionella* pneumonia continues to carry high mortality, particularly when diagnosis and effective treatment are delayed [4].

A central paradox motivates this review. *Legionella* is an aerobic Gram-negative intracellular bacterium that is exquisitely sensitive to fluoroquinolones and macrolides in vitro, and yet outcomes remain poor in severe disease. This discrepancy is increasingly attributed not to antimicrobial resistance but to diagnostic delay: *Legionella* does not grow on standard respiratory culture media; the most widely used rapid test (urinary antigen) detects only *L. pneumophila* serogroup 1 with variable sensitivity, which results in frequent false negatives in regions where non-serogroup 1 or non-pneumophila species predominate [5], and serology is retrospective. The consequence is that the true burden of *Legionella* pneumonia—including non-pneumophila species and non-serogroup-1 strains—is systematically underestimated [6], and severe disease caused by underrecognized species such as *Legionella longbeachae* is increasingly documented [7].

We argue that the field is now undergoing a diagnostic paradigm shift. Culture-independent molecular methods—metagenomic next-generation sequencing (mNGS), targeted NGS (tNGS), plasma microbial cell-free DNA (mcfDNA) sequencing, and syndromic multiplex PCR—are transforming the detection of *Legionella* and other fastidious pathogens [8,9,10], with direct implications for antimicrobial stewardship [11]. This shift, however, can only be exploited with a mechanistic understanding of why *Legionella* is so difficult to detect and treat, namely its intracellular lifestyle and host immune evasion.

This review is organized around one central theme: the management of *Legionella* pneumonia is transitioning from empirical, culture-dependent practice toward diagnosis-driven, precision care—a transition both enabled and constrained by the pathogen’s intracellular biology. We first establish this intracellular biology and its clinical-epidemiological context, then examine how culture-independent molecular diagnostics are reshaping detection, appraise contemporary treatment evidence, and synthesize these strands into a precision-management framework before closing with the field’s remaining limitations and knowledge gaps.

These strands are usually reviewed apart, and there is a reason to join them. The intracellular lifestyle is not background biology: it is what makes the organism invisible to culture and to a serogroup-restricted urinary antigen test, what makes β-lactams useless while intracellular-active agents work, and what shapes the inflammatory phenotype that clinicians interpret at the bedside. A diagnostic advance that is not tied to this biology is hard to interpret—an mNGS result only means something against a model of where the organism lives, how much of it is present, and what the host response has already suppressed. Integrating pathogenesis, epidemiology, diagnostics and treatment is therefore an argument about mechanism rather than a compilation of topics.

Several recent reviews have surveyed *Legionella* diagnosis [12], diagnosis and treatment [13], or global epidemiology and management [14], and the pathogenesis itself has been reviewed in detail elsewhere. This review differs in three respects. First, it treats diagnostic performance as a downstream consequence of intracellular biology rather than as a stand-alone comparison of platforms. Second, it places culture-independent methods—mNGS, tNGS, plasma cell-free DNA and syndromic PCR—in one frame with conventional tests, and states where pooled accuracy estimates exist and where they do not. Third, it grades the certainty behind therapeutic and preventive recommendations explicitly, including the places where those recommendations rest on indirect or observational evidence.

## 2. *Legionella pneumophila*: Virulence Factors and Intracellular Lifecycle

*Legionella pneumophila* is an environmental bacterium whose natural hosts are free-living amoebae; human disease is, in an evolutionary sense, accidental [15]. The same molecular machinery that allows *Legionella* to survive within amoebae is co-opted to replicate within human alveolar macrophages, making the intracellular lifecycle the foundation of pathogenesis [16]. Throughout this section we distinguish evidence obtained in murine models, in human cell lines, and in human infection, because the three carry different weight for clinical inference.

### 2.1. The Dot/Icm Type IV Secretion System

The defining virulence determinant of *L. pneumophila* is the Dot/Icm type IVB secretion system (T4BSS), a ~27-protein molecular machine that translocates an extraordinarily large repertoire of effector proteins into the host cell [17]. Recent cryo-electron tomography studies have resolved the in situ architecture of the Dot/Icm complex [18,19], revealing structural features distinct from the canonical T4SS and providing a molecular basis for its substrate specificity [20]; structural and functional characterization has further defined the assembly and regulation of this apparatus [21]. Substrate translocation is coordinated by dedicated chaperones, whose roles are only beginning to be redefined by structural studies [22], and effector gene expression is coupled to the nutritional status of the host cell through a nutrient-responsive LuxR-type regulator [23]. The system is essential for intracellular survival: peptidoglycan remodeling controls type IV secretion and intracellular growth [24], and the type IV pilin additionally promotes colonization [25].

### 2.2. The Legionella-Containing Vacuole and Effector Biology

Following phagocytosis, *Legionella* prevents phagolysosome fusion and remodels its phagosome into a specialized *Legionella*-containing vacuole (LCV) that recruits endoplasmic reticulum-derived vesicles through host Rab GTPases and the ER-shaping protein BAP31 [26], and is expanded by usurpation of host cell lipids [27]. Rab GTPase trafficking is manipulated at multiple levels, exemplified by the multi-tiered modulation of Rab10 dynamics [28] and the regulation of Rab5-dependent actin polymerization [29]. This remodeling is orchestrated by more than 300 translocated effectors (effector repertoires vary among strains)—a global atlas of predicted functional domains has cataloged their diversity [30], and a comprehensive two-hybrid analysis has begun to map the effector–effector and effector–host interactome [31]—which can be grouped into several functional classes.

A first class subverts host ubiquitin signaling and protein modification: the E3 ligase LegU1 modulates ubiquitination [32], the acetyltransferase Ceg10 inhibits host translation by targeting ribosomal protein S20 [33], and the adenylyltransferase SidL modifies host targets [34]; collectively, these effectors maintain host-cell ubiquitin homeostasis in a manner tailored to bacterial replication [35]. A distinct effector, BinA, subverts host amino-acid transport to sensitize TORC1 signaling [36]. Phosphoribosyl ubiquitination adds a further regulatory layer distinct from canonical ubiquitination [37].

**Figure 1:**
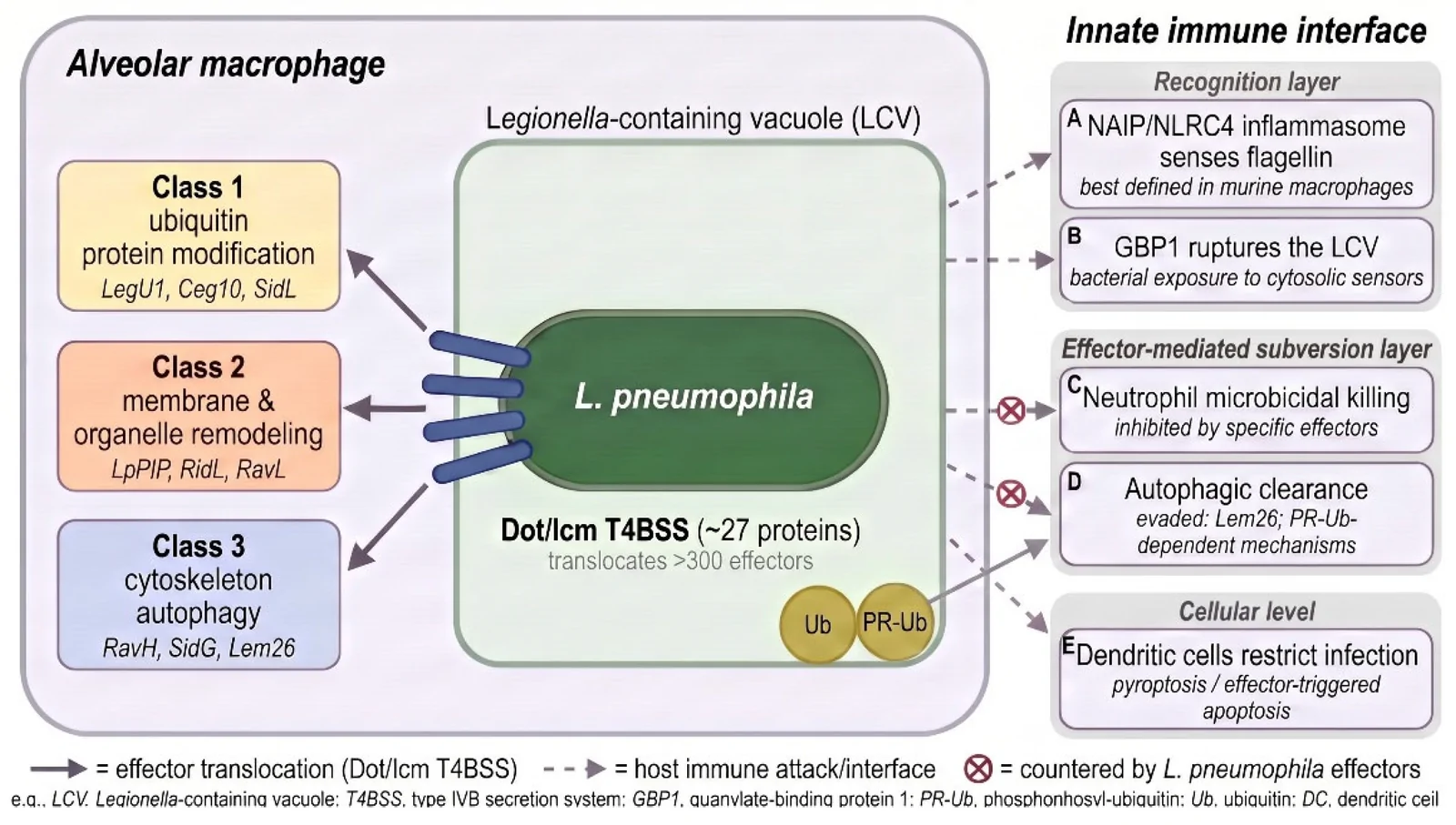
Dot/Icm-mediated effector biology and the innate immune interface of intracellular *Legionella pneumophila*. The Dot/Icm T4BSS (~27 proteins) translocates more than 300 effector proteins across the *Legionella*-containing vacuole (LCV) membrane into the host cytosol, where they act through three functional classes: ubiquitin and protein modification (Class 1: LegU1, Ceg10, SidL), membrane and organelle remodeling (Class 2: LpPIP, RidL, RavL), and cytoskeletal targeting with antagonism of autophagy (Class 3: RavH, SidG, Lem26). At the innate immune interface, flagellin is sensed by the NAIP/NLRC4 inflammasome, best defined in murine macrophages (A), and GBP1 mediates rupture of the LCV, exposing the bacterium to cytosolic sensors (B); specific effectors inhibit neutrophil microbicidal killing (C), autophagic clearance is evaded through Lem26 and phosphoribosyl-ubiquitin (PR-Ub)-dependent mechanisms (D), and dendritic cells restrict infection through pyroptosis and effector-triggered apoptosis (E). Solid arrows denote effector translocation; dashed arrows denote host immune attacks; ⊗ marks pathways countered by *L. pneumophila* effectors.

A second class remodels host membranes and organelles: phosphatidic acid is produced on the vacuole to recruit effectors [38], the effector LpPIP recruits protein phosphatase 1 to mitochondria [39], and the effectors RidL, which binds the large fission GTPase Drp1 [40], and RavL, a phosphoinositide 5-phosphatase that triggers mitochondrial fragmentation [41], each promote mitochondrial fission. A third class targets the host cytoskeleton—RavH through tandem WH2 domains [42] and SidG through the Arp2/3 complex [43]—while autophagy is antagonized by the ATG8-activated effector Lem26 [44] and additional phosphoribosyl-ubiquitination-dependent mechanisms [45]. Structural studies of individual effectors, including the glycosidase LegY [46] and the membrane-localized PelC and PelE effectors [47], are revealing the molecular basis of substrate recognition and providing templates for structure-guided inhibition. The functional redundancy of this effector arsenal—no single effector is individually essential—reflects the evolutionary arms race with the amoebal host and complicates both mechanistic dissection and therapeutic targeting, and partially explains the difficulty of translating single-effector inhibitors into clinical anti-*Legionella* therapies (Figure 1).

The functional redundancy of this arsenal is not only a technical inconvenience for dissecting virulence; it has three consequences that bear on the clinic. First, it explains why effector-directed inhibitors have not translated: an agent that neutralizes one enzyme leaves vacuole biogenesis largely intact, and the effector repertoire differs between strains and between species. Second, it shifts the plausible therapeutic surface from individual effectors to the nodes they converge on—ubiquitin homeostasis, phosphoinositide metabolism, mitochondrial dynamics, and the chaperone-dependent assembly of the Dot/Icm apparatus itself. Third, it offers a mechanistic reading of why the host response may underestimate bacterial burden: several effectors dampen the signaling that would otherwise amplify inflammation, so the inflammatory signal a clinician observes is the residue of a partly suppressed response rather than a read-out of bacterial load. These are inferences from mechanistic work rather than observations in patients, and none has yet been tested as a therapeutic strategy in clinical *Legionella* infection.

### 2.3. Host Immune Evasion and the Innate Immune Interface

*Legionella* engages and evades innate immunity at three successive levels. This layered evasion is often invoked to explain both the difficulty of diagnosis and the frequency of treatment failure, but the causal link has not been demonstrated in patients; it is better treated as a hypothesis that organizes the biology than as an established mechanism. First, at the level of recognition, flagellin is sensed by the NAIP/NLRC4 inflammasome, best defined in murine macrophages, with gasdermin-D and caspase-7 acting as key downstream substrates of pyroptotic cell death [48], and human guanylate-binding protein 1 (GBP1) facilitates rupture of the *Legionella*-containing vacuole to expose the bacterium to cytosolic sensors [49]. Second, at the level of effector-mediated subversion, neutrophil microbicidal responses are inhibited by specific effectors [50], the capsule of *L. longbeachae* favors intracellular replication and immune evasion [51], serogroup-1 lipopolysaccharide modulates host interaction [52], a collagen-like protein contributes to host-cell binding [53], and the bacterium evades host-autophagic clearance [45]. Third, at the level of the cellular host, dendritic cells restrict infection through pyroptosis and effector-triggered apoptosis [54], indicating that the balance between bacterial replication kinetics and host control—rather than the mere presence of immune recognition—determines outcome. This host–pathogen balance frames both the clinical heterogeneity of Legionnaires’ disease and the rationale for rapid, effective therapy: because the bacterium replicates intracellularly and actively suppresses the very responses that would otherwise contain it, delayed or ineffective treatment permits uncontrolled expansion, whereas prompt intracellular-active therapy tips the balance back toward host control. This intracellular niche dictates that antibiotics must penetrate macrophages and the LCV to exert bactericidal activity, which directly informs therapeutic selection and the urgency of early diagnosis. Most of the mechanistic evidence summarized in this section derives from murine macrophages and immortalized human cell lines; direct confirmation in human lung tissue or in patients is limited, and the model system is identified wherever it bears on interpretation.

Cytosolic recognition of Gram-negative bacteria extends beyond flagellin. ADP-heptose, an intermediate of lipopolysaccharide biosynthesis, is detected in the cytosol by the alpha-kinase ALPK1, which phosphorylates TIFA and assembles a TIFAsome that recruits TRAF6 and activates NF-κB [55,56]. In *Legionella* infection one of the best-documented cytosolic recognition events is different: NOD1 senses anhydro-disaccharide-tetrapeptide, a peptidoglycan fragment generated by the soluble lytic transglycosylase SltL, and the effector EnhC interferes with its production to dampen NF-κB activation [57]. Whether *Legionella*-derived ADP-heptose engages ALPK1 has, to our knowledge, not been demonstrated directly, and ALPK1 should therefore not be treated as the sole or even the principal sensor of *Legionella*: flagellin sensing by NAIP/NLRC4 [48], NOD1 sensing of peptidoglycan fragments [57], and GBP1-dependent vacuole rupture [49] act in parallel, and their relative weight is likely to depend on host cell type, strain and growth phase. The practical corollary is that no single innate sensor can serve as a diagnostic read-out of infection, and that effector-mediated interference acts downstream of, and in addition to, whatever recognition occurs.

### 2.4. Phosphatidylcholine at the Host–Pathogen Interface

One feature of the *Legionella* envelope is easy to overlook and clinically consequential: its dominant membrane-forming phospholipid is phosphatidylcholine (PC)—a lipid typical of eukaryotic membranes rather than of bacteria [58]. *Legionella* builds PC by two routes—methylation of phosphatidylethanolamine through PmtA and condensation of choline with CDP-diacylglycerol through PcsA—and it can also use exogenous choline. PC is not decorative. When PC synthesis is disabled, the Dot/Icm apparatus functions poorly, intracellular yields fall, and high-multiplicity cytotoxicity is lost, and the defect is corrected by restoring either biosynthetic route [59]. The lipid composition of the envelope is therefore part of the virulence program rather than a passive scaffold.

The host side of the same lipid is alveolar surfactant: surfactant is about 90% lipid by mass, and roughly 80% of that lipid is PC [58]. *L. pneumophila* secretes phospholipase A, which degrades surfactant phospholipids, releases lysophosphatidylcholine—a cytotoxic product—and raises the surface tension of surfactant in a pulsating bubble surfactometer [60]. This provides a mechanistic bridge between bacterial lipid metabolism and the alveolar injury that characterizes severe Legionnaires’ disease, and it places surfactant phospholipid handling within the host–pathogen conversation rather than at its margins. Because PC dominates the outer membrane, lipid composition is also a plausible determinant of permeability to antimicrobials and to membrane-active compounds, although to our knowledge antibiotic uptake has not been measured systematically as a function of membrane lipid composition in *Legionella*.

## 3. Clinical Spectrum and Epidemiology

### 3.1. Clinical Spectrum: From Pontiac Fever to Fatal Pneumonia

*Legionella* infection produces two clinically distinct syndromes. Pontiac fever is a self-limited, influenza-like illness without pneumonia, documented in outbreak settings among exposed cohorts [61]. The pathogenesis of Pontiac fever is not settled. The most frequently cited explanation is exposure to a high inoculum of non-viable or non-replicating organisms, which provokes an intense but transient inflammatory response without sustained intracellular replication; this account rests largely on outbreak investigations in which viable *legionellae* were not recovered from patients, and it has not been tested experimentally. Alternative explanations—a high-dose exposure with limited replication, or a host-response phenotype—have not been excluded. What is clear is that Pontiac fever resolves spontaneously and does not respond to antibiotics directed at replicating bacteria, which is consistent with, but does not prove, the non-viable-organism hypothesis. Legionnaires’ disease, in contrast, is a pneumonic illness that ranges from mild CAP to fulminant multilobar pneumonia with acute respiratory distress syndrome (ARDS), septic shock, and multiorgan failure; severe disease may require veno-venous extracorporeal membrane oxygenation [62]. Extrapulmonary manifestations—including rhabdomyolysis [63], myocarditis, and neurological features—are well described and can dominate the clinical presentation. Recent large cohorts have defined the clinical features and predictors of mortality in the modern era [64], while predictors of intensive care unit admission have been characterized separately [65], and rebound mortality after initial clinical improvement has been reported [66]. The principal host risk factors for severe disease are advanced age, immunosuppression—including hematological malignancy and transplantation—chronic lung disease, smoking, and male sex [67]. These host risk factors largely impair macrophage function and facilitate intracellular *Legionella* replication. Delayed initiation of appropriate therapy is the most consistently modifiable risk factor and directly links diagnostic failure to adverse outcome [4].

### 3.2. Epidemiology and Transmission

*Legionella* is acquired from environmental water sources—cooling towers, potable water systems, and premise plumbing—via aerosol inhalation. Human-to-human transmission is extremely rare and has not been well documented; particle-associated *Legionella* in drinking water represents a specific exposure route [68]. Outbreaks are linked to engineered water systems [69], including hospital cold-water clusters [70], and even household devices such as water flossers [71]. Healthcare-associated Legionnaires’ disease arising from hospital water systems is a persistent threat [72]. Meteorological factors influence water-system colonization and sporadic disease incidence [73]. Environmental transmission of *L. longbeachae* from potting soil [74] and seasonal host shifts in industrial cooling systems [75] illustrate the expanding ecological niche of this pathogen. The quantified burden is substantial and rising. In 2023, 28 EU/EEA countries reported 14,537 cases of Legionnaires’ disease, a notification rate of 3.2 per 100,000 population—the highest since EU-wide surveillance began and up from 2.6 per 100,000 in 2022—with France, Germany, Italy and Spain accounting for 72% of notifications; among 10,797 cases with a known outcome, 973 (9%) were fatal [76]. The species mix is regionally uneven and partly an artifact of testing: among EU/EEA travel-associated cases, 91% were reported as *L. pneumophila* serogroup 1 and a further 7% as *L. pneumophila* of unknown serogroup, whereas in New Zealand the dominant species shifted from *L. pneumophila* to *L. longbeachae* once PCR replaced serology as the main diagnostic method, and mean annual incidence rose from 1.6 to 3.9 per 100,000 between 2000–2009 and 2010–2020 [77]. Surveillance therefore measures what the local assay panel can see, and non-pneumophila disease is systematically under-counted where culture and urinary antigen testing dominate [78].

### 3.3. Prevention and Water-System Management

Because *Legionella* is acquired from engineered water systems rather than from person-to-person spread, prevention is fundamentally a water-management problem rather than an infection-control problem in the traditional sense. Surveillance of potable and premise plumbing water is the cornerstone of risk assessment, and quantitative methods such as Legiolert culture [79] and PCR-based risk evaluation [80] have improved detection of environmental colonization. Determinants of *Legionella* presence in hospital water systems have been characterized in multicenter surveillance studies, informing targeted rather than blanket intervention [72]. A range of control measures has been evaluated, including flushing protocols [81], alternative disinfection strategies in hot-water networks [82], and targeted blower-assisted thermal disinfection of persistently colonized distal outlets [83], with long-term control achievable through coordinated maintenance programs [84]. Quantitative microbial risk assessment and cost–benefit analysis now permit evidence-based selection of these interventions [85], and novel approaches such as water-vapor treatment are reshaping the microbial ecology of shower systems in healthcare facilities [86]. Integrating these environmental measures with clinical molecular diagnostics closes the loop from environmental source identification to patient outcome. The measures themselves are not equivalent in the strength of evidence behind them. Flushing addresses water age and stagnation and reduces colonization in controlled environmental studies, but the effect decays once flushing stops [81]. Silver-stabilized hydrogen peroxide has in vitro and field efficacy in hot-water networks, with limited persistence data [82]. Blower-assisted thermal disinfection clears persistently colonized distal outlets but does not treat the upstream system [83]. Water-vapor treatment reshapes the local shower microbiome and reduces exposure at the point of use [86]. Coordinated maintenance programs sustain control over years in single-system reports [84]. What is missing is the comparison a clinician would want: to our knowledge no randomized trial has used Legionnaires’ disease as an endpoint, almost all comparative data describe environmental colonization rather than human infection, and the strongest support for system-level control comes from water-safety-plan frameworks and quantitative risk assessment rather than from head-to-head trials of disinfectants [85]. Prevention evidence is therefore indirect throughout (Table 1).

## 4. The Diagnostic Paradigm Shift

As described above, diagnostic delay is the most important modifiable risk factor for poor outcomes in Legionnaires’ disease, and diagnostic performance therefore becomes the pivot on which *Legionella* outcomes turn, and it is here that the field has changed most rapidly.

### 4.1. Limitations of Conventional Diagnostics

Culture on buffered charcoal-yeast extract agar remains the reference standard and is the only method that supports species-level identification, serotyping, and outbreak typing [87], but it is slow, and its sensitivity is typically 60–80% in experienced laboratories and lower in routine practice, particularly after antimicrobial exposure [12], and interlaboratory comparison of culture- and PCR-based methods reveals further inconsistency [88]. The urinary antigen test is rapid but detects only *L. pneumophila* serogroup 1, with a performance assessment of five commercial tests confirming variable sensitivity [5]. In a meta-analysis of 21 studies and 5772 patients, pooled sensitivity was 0.79 (95% CI 0.71–0.85) and specificity 1.00 (95% CI 0.99–1.00), rising to 0.86 (0.78–0.91) when restricted to serogroup 1 [89]; cases undetected by repeated urinary antigen testing are well documented [90], and urinary antigen-negative *Legionella* pneumonia is common in critically ill patients [91]. These limitations mean that *Legionella* has historically been both underdiagnosed and, paradoxically, misattributed—often labeled “atypical pneumonia” and treated empirically without confirmation.

### 4.2. Molecular Methods: mNGS, tNGS, mcfDNA, and Syndromic PCR

Culture-independent molecular diagnostics have fundamentally changed this calculus. For PCR on respiratory specimens, a systematic review of 38 studies including 653 confirmed cases reported summary sensitivity of 97.4% (95% CI 91.1–99.2%) and specificity of 98.6% (97.4–99.3%) [92]; these figures are almost certainly optimistic, because many included studies used case–control designs and a composite reference standard that itself incorporated PCR. Metagenomic NGS enables unbiased detection of *Legionella* directly from lower respiratory tract specimens, including in culture-negative and serogroup-1-negative cases [93], and is increasingly deployed as a routine service in intensive care settings [8]; fatal cases diagnosed only by mNGS illustrate its clinical value [94]. Targeted NGS and hybrid-capture approaches offer enhanced sensitivity for respiratory pathogens with faster turnaround [10], with clinical application demonstrated in severe pneumonia [95] and in specific *Legionella* case identification [96]. Plasma microbial cell-free DNA sequencing detects a broad pathogen spectrum from a minimally invasive blood sample, including *Legionella micdadei* endocarditis [97]. Rapid CRISPR-based assays [98] and PCR melting-curve discrimination [99] further expand the toolkit, and species-level detection beyond *L. pneumophila* is increasingly feasible [6]. Syndromic multiplex PCR panels deliver same-day results and improve diagnostic yield and antimicrobial stewardship in CAP when used judiciously [11]. The principal diagnostic methods and their comparative characteristics are summarized in Table 2.

Comparative accuracy depends on more than the platform. Specimen type comes first: lower respiratory specimens—bronchoalveolar lavage fluid, bronchial aspirate or an adequate sputum sample—carry far more bacterial DNA than upper-airway swabs, so a negative result from a poor specimen is uninformative. Prior antimicrobial exposure comes second: culture positivity falls steeply once effective intracellular-active agents have been given, whereas nucleic-acid detection is much less affected, so a positive PCR after antibiotics may be the only evidence of the pathogen while a negative culture does not exclude it [12]. Pathogen burden comes third: in mild or early disease, and in non-pneumophila infection, genome copy numbers can fall below the limit of detection of any assay. Contamination is the counterweight: because mNGS and tNGS read all nucleic acid present, reagent and environmental contamination and upper-airway flora generate signals that must be weighed against pre-test probability, and a single low-abundance read without clinical and radiological corroboration should not drive therapy [8,9]. None of these modifiers is captured by a single sensitivity figure, which is why the pooled estimates in Table 2 are reported with their provenance rather than as a league table.

Sensitivity and specificity are properties of the assay, but the figures a clinician acts on—the positive and negative predictive values—are properties of the assay and the patient together. Because the prevalence of *Legionella* among patients tested for pneumonia varied widely across the included studies, from a few percent in unselected community-acquired pneumonia to more than 20% in outbreak settings and in intensive-care cohorts selected for severity, a single pooled predictive value would mislead. The source meta-analyses therefore report sensitivity and specificity only, and we do not pool predictive values here. For illustration, at a pre-test probability of 5%, the pooled urinary antigen estimates (sensitivity 0.79) with a specificity of 0.99—the lower confidence bound rather than the point estimate of 1.00—correspond to a positive predictive value of about 0.81 and a negative predictive value of about 0.99. This is the arithmetic behind the argument that a negative antigen test in a low-prevalence population carries little information, whereas a positive one carries a great deal.

### 4.3. Diagnostic Stewardship and the Path to Precision Management

**Figure 2:**
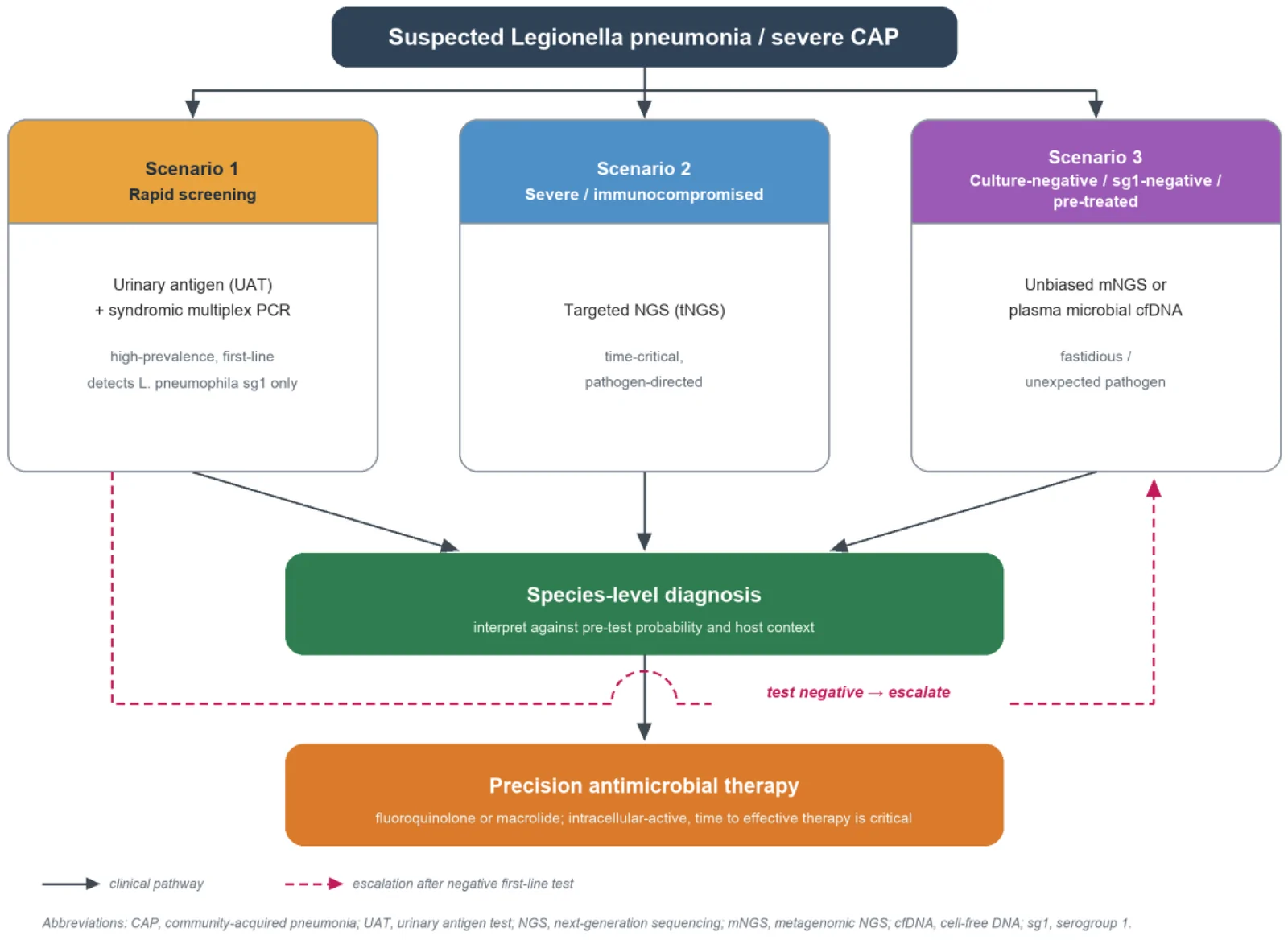
Diagnostic stewardship pathway for suspected *Legionella* pneumonia, from clinical suspicion to precision therapy. This framework serves as a clinical decision aid rather than a formal clinical guideline. Three clinical scenarios map to distinct molecular strategies: rapid urinary antigen testing and syndromic multiplex PCR for high-prevalence screening (Scenario 1), targeted next-generation sequencing for severe or immunocompromised-host pneumonia where time to pathogen-directed therapy is critical (Scenario 2), and unbiased metagenomic next-generation sequencing or plasma microbial cell-free DNA for culture-negative, serogroup-1-negative, or antibiotic-pretreated cases (Scenario 3); negative first-line results escalate to broader testing (dashed arrow). Results are interpreted against pre-test probability and host context to reach species-level diagnosis and precision antimicrobial therapy. Abbreviations: CAP, community-acquired pneumonia; UAT, urinary antigen test; NGS, next-generation sequencing; tNGS, targeted NGS; mNGS, metagenomic NGS; cfDNA, cell-free DNA; sg1, serogroup 1.

The promise of these tools is realized only through diagnostic stewardship—the selection of the right test for the right patient at the right time. Uncritical deployment of broad mNGS carries its own hazards: because these assays detect nucleic acids from background and commensal organisms, positive results must be interpreted against pre-test probability and the host context, and false-positive signals can drive unnecessary or inappropriate therapy. The decision of which modality to apply therefore depends on the clinical scenario: urinary antigen and syndromic panels for rapid, high-prevalence screening; targeted NGS for severe or immunocompromised-host pneumonia where time to pathogen-directed therapy is critical [95]; and unbiased mNGS or plasma cell-free DNA for culture-negative, serogroup-1-negative, or antibiotic-pretreated cases in which a fastidious or unexpected pathogen is suspected [97]. We argue that *Legionella*, as a fastidious intracellular pathogen with a limited repertoire of effective antimicrobial agents, is precisely the kind of target for which culture-independent molecular diagnostics confer the greatest clinical benefit—but only when the right test is matched to the right patient through explicit diagnostic stewardship (Figure 2).

To make this operational we set out explicit rules rather than principles (Table 3). Three decisions need criteria: whom to test, how to escalate after a negative first-line result, and how to read a discordant result. We test when pre-test probability is high enough to change management—severe CAP requiring intensive care, immunosuppression, a compatible exposure history, hyponatremia with gastrointestinal symptoms and otherwise unexplained illness, or failure of β-lactam therapy—and we do not test in mild CAP where the result would not alter a macrolide-based plan. We escalate after a negative urinary antigen test when clinical probability remains high, when antibiotics were given before sampling, when the patient is immunocompromised, or when non-serogroup-1 or non-pneumophila disease is plausible; in those situations a lower respiratory specimen for PCR or tNGS is more informative than repeating the antigen test. We read discordance explicitly: a positive urinary antigen test with a negative PCR usually indicates true serogroup-1 disease, and no further testing is needed; a positive PCR with a negative culture is expected when antibiotics preceded sampling; and a low-abundance mNGS signal in a patient improving on non-*Legionella* therapy is more likely contamination than disease.

## 5. Treatment Optimization

### 5.1. Antibacterial Therapy

*Legionella* is intrinsically resistant to β-lactams owing to β-lactamase-mediated inactivation [100]; effective agents are those that achieve high intracellular concentrations. As described in Section 2, *Legionella* replicates within the LCV of alveolar macrophages; antibiotics must penetrate macrophages and vacuolar membranes to reach active concentrations. Principal options include fluoroquinolones (levofloxacin, moxifloxacin) and macrolides (azithromycin). Contemporary comparative data are entirely observational. A propensity-matched cohort found broadly similar outcomes for levofloxacin and azithromycin, but matching balances only measured confounders, and severity, time to effective therapy and the specific macrolide used were not randomly assigned [101]. A large administrative analysis associated regimen with hospital mortality in severe cases, yet administrative data cannot separate drug choice from illness severity or from the decision to escalate care [102], and single-center retrospective series add little beyond these two [103]. The two data sources also differ in case mix: a hospital cohort and a national administrative database of hospitalizations do not sample the same patients, and neither adjusted for *Legionella*-specific severity scores. The defensible summary is that fluoroquinolones and azithromycin appear similar in moderate disease, that no randomized comparison exists, and that the severe-disease evidence is too confounded to support a firm preference. Time to effective therapy is a consistent and modifiable predictor of mortality [4], underscoring the importance of early, *Legionella*-covering empirical therapy in severe CAP [104].

### 5.2. Combination Therapy, Duration, and Adjunctive Therapy

Whether combination therapy improves outcomes in severe *Legionella* pneumonia is unresolved. A multicenter retrospective study did not consistently demonstrate superiority over monotherapy, but treatment was chosen by the treating team, so confounding by indication is likely and the confidence interval around any benefit is wide [105]. The same caution applies to duration: recommended courses rest on pharmacokinetic reasoning and observational data rather than on randomized comparison, and no trial has tested shortening therapy in *Legionella* specifically. Current guidelines recommend either a fluoroquinolone (levofloxacin 750 mg daily or moxifloxacin 400 mg daily) or azithromycin (500 mg daily) as first-line therapy, with combination therapy reserved for severe or refractory disease and immunosuppressed hosts [1]. Adjunctive corticosteroid therapy in severe CAP remains debated: in the REMAP-CAP corticosteroid domain (*n* = 658), a 7-day course of hydrocortisone was stopped for futility and judged unlikely to confer a large mortality reduction in severe CAP [106], whereas the larger CAPE COD trial (795 patients analyzed) reported lower 28-day mortality with hydrocortisone (6.2% vs. 11.9%; *p* = 0.006) [107]. Both trials enrolled patients with all-cause severe CAP; neither reported a *Legionella*-specific subgroup analysis, so the effect in *Legionella* pneumonia is unknown, and extrapolating a pooled all-cause estimate to this pathogen is an assumption rather than an inference. Newer agents, including delafloxacin with intracellular activity [108] and omadacycline in severe disease [109,110], expand the therapeutic armamentarium. Emergent azithromycin resistance mechanisms [111] and reduced susceptibility in clinical isolates [112] warrant surveillance, and standardization of susceptibility testing remains an open need [113]. Key treatment comparisons and their evidence are summarized in Table 4.

## 6. Toward Precision Management

The convergence of intracellular pathogenesis, molecular diagnostics, and evolving treatment evidence points toward a precision-management paradigm for *Legionella* pneumonia, replacing empirical, often delayed, and sometimes incorrect approaches. Empirical antibiotic treatment for CAP frequently fails to cover *Legionella* adequately [114], and accuracy is improved by molecular diagnosis [115]. Beyond antimicrobials, the intracellular niche itself is emerging as a therapeutic target: *Legionella* subverts the host endocytic pathway [116], modulates macrophage function through epigenetic reprogramming [117], relies on copper resistance for environmental and host survival [118], and its environmental antimicrobial susceptibility is shifting [119]. These host-directed strategies remain preclinical and are not yet ready for routine clinical application.

## 7. Limitations

**Figure 3:**
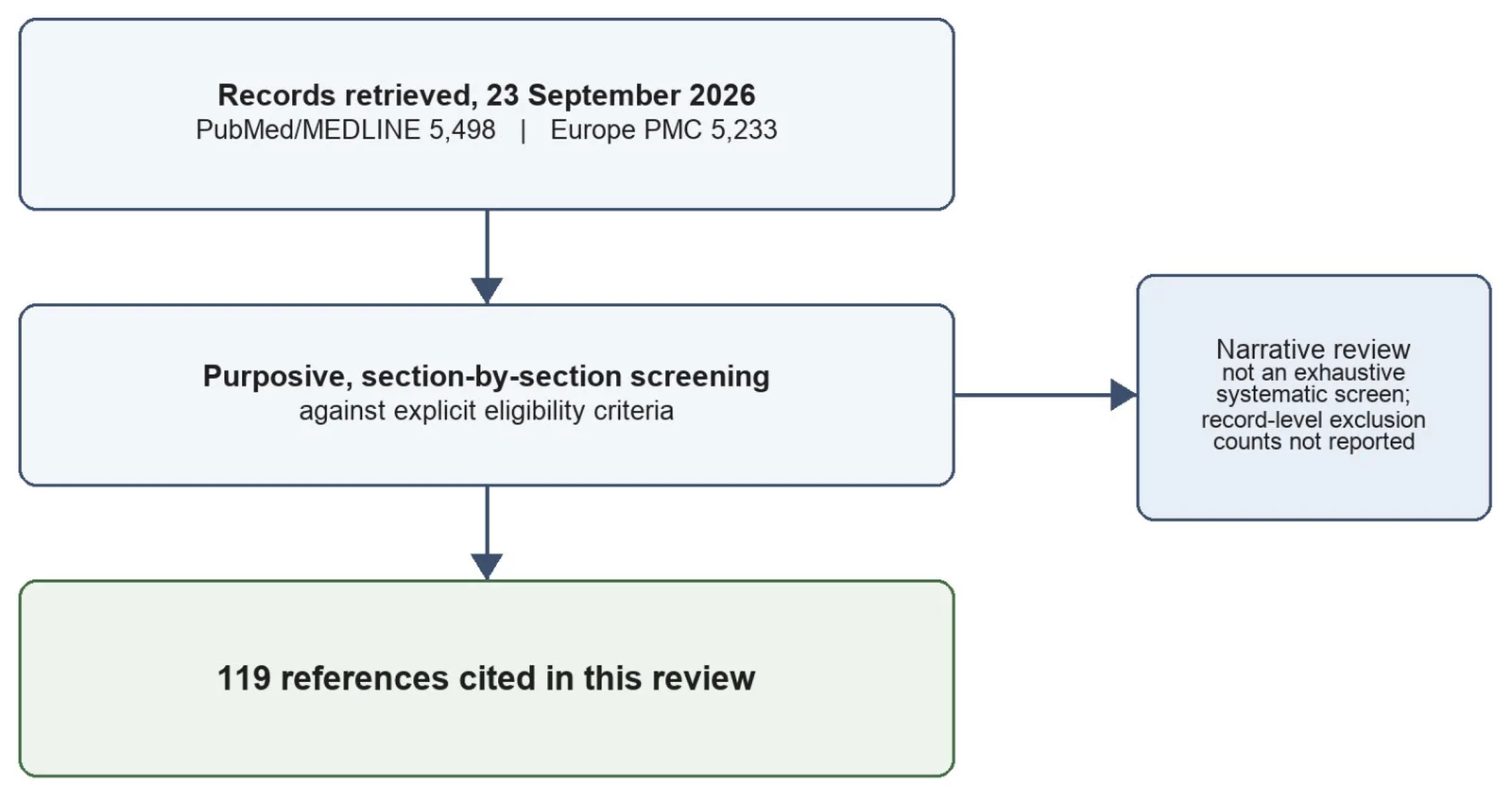
Retrieval and selection of the literature. The PubMed/MEDLINE and Europe PMC searches of 23 September 2026 returned 5498 and 5233 records, respectively. Because this is a narrative review, selection was purposive and organized by section rather than exhaustive, so record-level exclusion counts are not reported; 119 references are cited in this review.

This review has several limitations. It is a narrative rather than a systematic review. We report the search strings, eligibility criteria, retrieval counts and selection procedure (Figure 3); because selection was purposive rather than exhaustive, record-level exclusion counts are not reported. The selection of studies was not governed by a prospectively registered protocol, risk of bias was not formally assessed study by study, and quantitative synthesis was not performed. We also did not systematically search gray literature, which may introduce publication bias. The literature on the molecular diagnosis of *Legionella* pneumonia is rapidly evolving and is dominated by observational and single-center studies. These single-center studies are constrained by local case-mix and assay platforms, limiting generalizability of the reported diagnostic performance and clinical outcomes. Finally, because *Legionella* pneumonia is relatively uncommon, much of the severe-disease evidence derives from case series and retrospective cohorts rather than randomized trials, and the comparative effectiveness of diagnostic strategies has not been established prospectively.

## 8. Future Directions and Conclusions

Several knowledge gaps remain. First, the clinical impact of non-pneumophila *Legionella* species and non-serogroup-1 strains is poorly quantified, and species-level diagnosis is rarely achieved [6]. Second, prospective data on whether early molecular diagnosis translates into mortality benefit are lacking. Third, the role of host-directed therapies—modulating the intracellular niche rather than targeting the bacterium—is largely unexplored, although the effector repertoire provides a mechanistic foundation [30]. Such host-directed approaches remain preclinical and require further in vivo validation before clinical translation. Fourth, the expansion of *Legionella*’s environmental niche under climate change raises unresolved questions about prevention and surveillance [73].

Addressing these gaps will require multicenter studies linking molecular diagnostics to outcomes, species-resolved epidemiological surveillance, and mechanistic work on host-directed strategies. Until such studies exist, the framework proposed here should be read as a hypothesis about how to organize care rather than as evidence that reorganizing care improves outcomes: neither the clinical benefit nor the cost-effectiveness of a diagnosis-driven pathway has been demonstrated prospectively, and the added laboratory cost of broad molecular testing is itself a reason to require that demonstration. *Legionella* pneumonia is at once an old disease and a new frontier of diagnostic and therapeutic precision; closing the gap between detection and management is the central challenge for the next decade.

## 9. Search Strategy and Selection Criteria

We searched PubMed/MEDLINE through the NCBI E-utilities and Europe PMC on 23 September 2026, without language restriction, using the following reproducible strings. Both strings were restricted to the title and abstract fields so that the two retrievals are directly comparable; the Europe PMC search was further limited to MEDLINE and PMC records, excluding patents and preprints.

PubMed: (“*Legionella*”[MeSH Terms] OR *Legionella*[tiab] OR “Legionnaires disease”[tiab]) AND (pneumonia[tiab] OR diagnosis[tiab] OR “type IV secretion”[tiab] OR “Dot/Icm”[tiab] OR metagenomic[tiab] OR “next-generation sequencing”[tiab] OR “urinary antigen”[tiab] OR treatment[tiab] OR macrolide*[tiab] OR fluoroquinolone*[tiab])-5498 records.

Europe PMC: (TITLE_ABS:“*Legionella*” OR TITLE_ABS:“Legionnaires disease”) AND (TITLE_ABS:“pneumonia” OR TITLE_ABS:“diagnosis” OR TITLE_ABS:“type IV secretion” OR TITLE_ABS:“Dot/Icm” OR TITLE_ABS:“metagenomic” OR TITLE_ABS:“next-generation sequencing” OR TITLE_ABS:“urinary antigen” OR TITLE_ABS:“treatment” OR TITLE_ABS:“macrolide” OR TITLE_ABS:“fluoroquinolone”) AND (SRC:MED OR SRC:PMC)-5233 records.

Records were eligible if they reported original clinical, epidemiological, diagnostic or mechanistic data on *Legionella* infection in humans or in relevant experimental systems, or were systematic reviews or society guidelines bearing on diagnosis or management. Conference abstracts, editorials, letters without original data, and non-peer-reviewed material were excluded.

The strings are deliberately broad, and neither retrieval is a defined evidence set that could be screened record by record by two reviewers. This is a narrative review, and selection was purposive and organized by section: for each section, records returned by the strings were read in order of relevance and supplemented by the reference lists of included articles, and the most informative primary studies, systematic reviews and guidelines were retained. We therefore report retrieval counts rather than PRISMA-style exclusion counts, which would imply a systematic screen that we did not perform. One cited source is not a database record: the ECDC annual epidemiological report, which is official surveillance data and is the only reference in this review that is not indexed in either database. Figure 3 summarizes the retrieval and the selection procedure.

## Figures and Tables

**Table 1:** Water-system control measures: target, evidence available, and certainty.

Measure	Target	Evidence Available	Reported Outcome	Certainty
Flushing and water-age management	Stagnation in premise plumbing	Controlled environmental studies [81]	Lower colonization while flushing continues	Low (surrogate endpoint)
Silver-stabilized hydrogen peroxide	Hot-water network biofilm	In vitro and field evaluation [82]	Reduced *L. pneumophila*; persistence data limited	Low
Blower-assisted thermal disinfection	Persistently colonized distal outlets	Single-center intervention [83]	Clearance at treated outlets	Low
Water-vapor treatment	Shower-system microbiome	Microbiome intervention study [86]	Reduced exposure at the point of use	Low
Coordinated maintenance program	Whole water system	Long-term single-system report [84]	Sustained control over years	Low-moderate
Quantitative risk assessment and cost–benefit analysis	Selection among interventions	Economic modeling [85]	Ranking of measures by cost-effectiveness	Low

No randomized trial has used Legionnaires’ disease as an endpoint. Outcomes for most measures are environmental colonization or point-of-use exposure rather than human infection, which is why certainty is graded low throughout.

**Table 2:** Comparison of diagnostic methods for *Legionella* pneumonia, with reported accuracy where a pooled estimate exists.

Method	Principle	Turnaround	Reported Accuracy	Key Limitations
Culture (BCYE agar)	Lower respiratory tract; isolation of viable organism; reference standard	3–10 days	Sensitivity 60–80%; specificity ~100% [12]	Slow; specialized media; prior antibiotics reduce yield
Urinary antigen	Urine; *L. pneumophila* serogroup 1 lipopolysaccharide	<1 h	Pooled sensitivity 0.79 (95% CI 0.71–0.85), specificity 1.00 (0.99–1.00); serogroup 1 sensitivity 0.86 (0.78–0.91) [89]	Serogroup 1 only; false negatives in non-serogroup-1 and non-pneumophila disease
Serology	Paired sera; antibody response	Weeks	Retrospective only	No acute diagnostic value
PCR (respiratory)	Sputum, BAL fluid, bronchial aspirate; nucleic acid amplification	Hours	Summary sensitivity 97.4% (91.1–99.2%), specificity 98.6% (97.4–99.3%) [92]; likely overestimated	Specimen quality; contamination; primer coverage
Syndromic multiplex PCR	Respiratory panel	Hours	No pooled estimate for *Legionella* specifically; panel-dependent yield, with improved yield and stewardship in CAP [11]	Panel composition; not all species covered
Metagenomic NGS	Unbiased sequencing of all nucleic acids	24–72 h	No pooled estimate; case-level evidence [93,94]	Background and commensal signal; interpretation; cost
Targeted NGS/hybrid capture	Enriched sequencing of the lower respiratory tract	24–48 h	No pooled estimate; single-study yields [10,95]	Panel-limited; cost
Plasma microbial cfDNA	Blood; cell-free DNA sequencing	1–2 days	No pooled estimate; case reports [97]	Low burden in localized infection; cost

Accuracy figures are those of the cited meta-analyses and apply to the populations and reference standards used in them; culture and PCR estimates carry different degrees of verification bias. No pooled estimate exists for the sequencing-based methods, for which single-study yields are cited. Turnaround times reflect typical ranges reported in the literature cited in this review.

**Table 3:** Explicit criteria for *Legionella* testing, escalation after a negative result, and interpretation of discordant results.

Decision	Explicit Criteria
Whom to test	Severe CAP (intensive care, or PSI class IV-V); immunosuppression; compatible exposure (cooling tower, potable water, travel, potting soil); hyponatremia with gastrointestinal symptoms otherwise unexplained; failure of β-lactam therapy.
Whom not to test	Mild community-acquired pneumonia in which a positive result would not change a macrolide-based plan; patients in whom a *Legionella* result would not alter management.
First-line test	Urinary antigen test plus a lower respiratory specimen for PCR where one can be obtained; culture in parallel when species-level identification or outbreak typing matters.
Escalation after a negative result	Persistent high clinical probability; antibiotics before sampling; immunocompromise; suspected non-serogroup-1 or non-pneumophila species; negative antigen and PCR results with ongoing severe pneumonia lead to tNGS or mNGS on a lower respiratory specimen; plasma cfDNA when no respiratory specimen can be obtained.
Do not	Repeat a negative urinary antigen test; use mNGS as the first test in mild CAP; treat a low-abundance mNGS signal without clinical corroboration.
Urinary antigen positive, PCR negative	Treat as serogroup-1 disease; further testing adds nothing.
PCR positive, culture negative	Expected when antibiotics preceded sampling; treat, and do not repeat culture for confirmation.
mNGS positive, all other tests negative	Weigh organism abundance, biological plausibility and host status; do not escalate therapy on a single low-abundance read.

The criteria are a decision aid derived from the evidence reviewed here, not a formally validated or prospectively tested algorithm.

**Table 4:** Summary of treatment evidence in *Legionella* pneumonia, with certainty of evidence.

Comparison	Study Design	Outcome Assessed (Sample Size, Where Reported)	Key Finding	Certainty	Reference (s)
Levofloxacin vs. azithromycin	Propensity-matched cohort	Clinical efficacy	Broadly similar outcomes	Low (observational; measured confounders only)	[101]
Azithromycin vs. fluoroquinolones	Retrospective cohorts	Clinical outcomes	Similar; some favor fluoroquinolones in severe disease	Very low	[103]
Regimen and hospital mortality	Retrospective cohort using administrative data	Hospital mortality	Regimen associated with mortality in severe disease	Very low (confounding by indication)	[102]
Monotherapy vs. combination	Multicenter retrospective cohort	Clinical outcomes	No consistent superiority of combination therapy	Very low	[105]
Adjunctive hydrocortisone	RCTs in all-cause severe CAP: REMAP-CAP (*n* = 658), CAPE COD (*n* = 795)	90-day and 28-day mortality	REMAP-CAP stopped for futility; CAPE COD reduced 28-day mortality (6.2% vs. 11.9%)	Moderate for all-cause CAP; not established for *Legionella* (no subgroup analysis)	[106,107]
Delafloxacin	In vitro intracellular model	Intracellular activity	Active against intracellular *Legionella*	Very low (preclinical)	[108]
Omadacycline	Case series	Clinical response	Success in severe disease	Very low	[109,110]

Certainty is graded qualitatively by design (randomized > prospective cohort > retrospective cohort > case series > preclinical). Randomized evidence exists only for adjunctive corticosteroid therapy in all-cause severe CAP, and neither trial reported a *Legionella*-specific subgroup analysis.

## Data Availability

This is a narrative review; no new data were generated or analyzed. All data supporting this review are derived from the published literature cited herein.

## References

[1] Martin-Loeches I., Torres A., Nagavci B., Aliberti S., Antonelli M., Bassetti M., Bos L.D., Chalmers J.D., Derde L., de Waele J. (2023). ERS/ESICM/ESCMID/ALAT guidelines for the management of severe community-acquired pneumonia. Intensive Care Med..

[2] Fraser D.W., Tsai T.R., Orenstein W., Parkin W.E., Beecham H.J., Sharrar R.G., Harris J., Mallison G.F., Martin S.M., E McDade J. (1977). Legionnaires’ disease: Description of an epidemic of pneumonia. N. Engl. J. Med..

[3] McDade J.E., Shepard C.C., Fraser D.W., Tsai T.R., Redus M.A., Dowdle W.R. (1977). Legionnaires’ disease: Isolation of a bacterium and demonstration of its role in other respiratory disease. N. Engl. J. Med..

[4] Pulsipher A.M., Khattar G., VanDolah H., Gotway M., Ham K., Patel R., Simner P.J., Vikram H.R., Sen A., Skolnik A. (2026). Time to effective therapy and mortality in community-acquired *Legionella* pneumonia: A multicenter retrospective cohort study. Ann. Am. Thorac. Soc..

[5] Azzato F., Elieh L., Brizuela R., Hor E., Raios K., Williams E., Lim C.K., Graham M. (2026). Performance assessment of five *Legionella* urinary antigen tests for *Legionella pneumophila* and *Legionella longbeachae*. Pathology.

[6] Riku M., Nakamura R., Terashima T., Sakanashi D., Nakata S., Kawamura M., Ohnishi K., Ito H., Watanabe E., Mikamo H. (2024). Detection of *Legionella* species other than *Legionella pneumophila* in formalin-fixed paraffin-embedded tissue: An autopsy case study. Pathol. Int..

[7] Shinomiya S., Tanaka T., Shionoya I., Mura T., Nakase K., Takahara Y., Oikawa T., Mizuno S. (2023). A case of severe pneumonia caused by *Legionella longbeachae* with positive results by a *Legionella* urinary antigen detection kit. Ther. Adv. Infect. Dis..

[8] Charalampous T., Alcolea-Medina A., Snell L.B., Alder C., Tan M., Williams T.G.S., Al-Yaakoubi N., Humayun G., Meadows C.I.S., Wyncoll D.L.A. (2024). Routine metagenomics service for ICU patients with respiratory infection. Am. J. Respir. Crit. Care Med..

[9] Park S.Y., Chang E.J., Ledeboer N., Messacar K., Lindner M.S., Venkatasubrahmanyam S., Wilber J.C., Vaughn M.L., Bercovici S., Perkins B.A. (2023). Plasma microbial cell-free DNA sequencing from over 15,000 patients identified a broad spectrum of pathogens. J. Clin. Microbiol..

[10] Yin Y., Zhu P., Guo Y., Li Y., Chen H., Liu J., Sun L., Ma S., Hu C., Wang H. (2024). Enhancing lower respiratory tract infection diagnosis: Implementation and clinical assessment of multiplex PCR-based and hybrid capture-based targeted next-generation sequencing. eBioMedicine.

[11] Markussen D.L., Serigstad S., Ritz C., Knoop S.T., Ebbesen M.H., Faurholt-Jepsen D., Heggelund L., van Werkhoven C.H., Clark T.W., Bjørneklett R.O. (2024). Diagnostic stewardship in community-acquired pneumonia with syndromic molecular testing: A randomized clinical trial. JAMA Netw. Open.

[12] Bai L., Yang W., Li Y. (2023). Clinical and Laboratory Diagnosis of *Legionella* Pneumonia. Diagnostics.

[13] Viasus D., Gaia V., Manzur-Barbur C., Carratalà J. (2022). Legionnaires’ Disease: Update on Diagnosis and Treatment. Infect. Dis. Ther..

[14] Ma J., Dong T., Li F., He C., Li B., Liu Z. (2026). Legionellosis: Global Epidemiology and Current Perspectives on Diagnosis and Treatment. Infect. Drug Resist..

[15] Moreno A.B., Paranjape K., Cederblom M., Kay E., Dobre-Lereanu C., Andersson D.I., Guy L. (2025). Host-specific adaptation of *Legionella pneumophila* to single and multiple hosts. Mol. Biol. Evol..

[16] Leenheer D., Moreno A.B., Paranjape K., Murray S., Jarraud S., Ginevra C., Guy L. (2023). Rapid adaptations of *Legionella pneumophila* to the human host. Microb. Genom..

[17] Lockwood D.C., Amin H., Costa T.R.D., Schroeder G.N. (2022). The *Legionella pneumophila* Dot/Icm type IV secretion system and its effectors. Microbiology.

[18] Yue J., Heydari S., Park D., Chetrit D., Tachiyama S., Guo W., Botting J.M., Wu S., Roy C.R., Liu J. (2025). In situ structures of the *Legionella* Dot/Icm T4SS identify the DotA-IcmX complex as the gatekeeper for effector translocation. Proc. Natl. Acad. Sci. USA.

[19] Dutka P., Liu Y., Maggi S., Ghosal D., Wang J., Damke P.P., Carter S.D., Zhao W., Vijayrajratnam S., Vogel J.P. (2026). In situ molecular organization and heterogeneity of the *Legionella* Dot/Icm T4SS. FEBS Lett..

[20] Durie C.L., Sheedlo M.J., Chung J.M., Byrne B.G., Su M., Knight T., Swanson M., Lacy D.B., Ohi M.D. (2020). Structural analysis of the *Legionella pneumophila* Dot/Icm type IV secretion system core complex. eLife.

[21] Roberts J.R., Frick-Cheng A.E., Styron H.J., Durie C.L., Chang L., Ohi M.D. (2025). The *Legionella pneumophila* Dot/Icm type IV secretion system is structurally and functionally resilient in absence of species-specific proteins Dis2 and Dis3. J. Mol. Biol..

[22] Vijayrajratnam S., Hasanovic A., Vogel J.P. (2026). Redefining the role of chaperones for Dot/Icm T4BSS-mediated substrate translocation by *Legionella pneumophila*. Cell Rep..

[23] Adler C., Segal G. (2026). A nutrient-responsive LuxR regulator orchestrates effector gene expression across the *Legionella* genus. Mol. Microbiol..

[24] Boamah D., Gilmore M.C., Bourget S., Ghosh A., Hossain M.J., Vogel J.P., Cava F., O’connor T.J. (2023). Peptidoglycan deacetylation controls type IV secretion and the intracellular survival of the bacterial pathogen *Legionella pneumophila*. Proc. Natl. Acad. Sci. USA.

[25] Barajas A., Mayoral J., Cianciotto N.P. (2026). Major type IV pilin of *Legionella pneumophila* enhances bacterial iron acquisition/assimilation, independently of its role in piliation. mBio.

[26] Chadha A., Yanai Y., Oide H., Wakana Y., Inoue H., Saha S., Paul M., Tagaya M., Arasaki K., Mukherjee S. (2024). *Legionella* uses host Rab GTPases and BAP31 to create a unique ER niche. Cell Rep..

[27] Ghosh S., Bandyopadhyay S., Smith D.M., Adak S., Semenkovich C.F., Nagy L., Wolfgang M.J., O’connor T.J. (2024). *Legionella pneumophila* usurps host cell lipids for vacuole expansion and bacterial growth. PLoS Pathog..

[28] Kubori T., Arasaki K., Oide H., Kitao T., Nagai H. (2024). Multi-tiered actions of *Legionella* effectors to modulate host Rab10 dynamics. eLife.

[29] Saraiva J., Bugalhão J.N., Carvalho T., Hensel Z., Crevenna A., Mota L.J., Franco I.S. (2026). The small GTPase Rab5 inhibits actin polymerization mediated by the *Legionella pneumophila* effector VipA. Med. Microbiol. Immunol..

[30] Patel D.T., Stogios P.J., Jaroszewski L., Urbanus M.L., Sedova M., Semper C., Le C., Takkouche A., Ichii K., Innabi J. (2025). Global atlas of predicted functional domains in *Legionella pneumophila* Dot/Icm translocated effectors. Mol. Syst. Biol..

[31] Mount H.O., Urbanus M.L., Sheykhkarimli D., Coté A.G., Laval F., Coppin G., Kishore N., Li R., Spirohn-Fitzgerald K., Petersen M.O. (2024). A comprehensive two-hybrid analysis to explore the *Legionella pneumophila* effector-effector interactome. mSystems.

[32] Zhao B., He S., Huang M., Lu Y., Ge Z. (2025). *Legionella pneumophila* effector protein LegU1 mediates the ubiquitination of endoplasmic reticulum chaperone BiP. J. Microbiol. Biotechnol..

[33] Gao J., Xu W., Zhou Z., Lu L., Ge Z., Wang W., Lu Y., Ge H. (2026). *Legionella* effector Ceg10 acetylates RPS20 to inhibit host translation and induce cell cycle arrest. Proc. Natl. Acad. Sci. USA.

[34] Black J.J., Burroughs A.M., Rashan E.H., Nosal C.R., Romanov K.A., Catipovic M.A., Valadez C., Aravind L., Hicks K.G., Meyers C.L.F. (2026). The *Legionella pneumophila* effector SidL is an adenylyltransferase that modifies the glycolytic intermediate 3-phosphoglycerate. Mol. Cell.

[35] Fu J., Li S., Guan H., Li C., Zhao Y.B., Chen T.T., Xian W., Zhang Z., Liu Y., Guan Q. (2024). *Legionella* maintains host cell ubiquitin homeostasis by effectors with unique catalytic mechanisms. Nat. Commun..

[36] Circu M., Castore R., Latimer B., Shames S., Roy C.R., Dragoi A.M., Ivanov S.S. (2026). The *Legionella pneumophila* type IVb secretion system effector BinA subverts amino acid transport to sensitize TORC1 signaling in macrophages. PLoS Pathog..

[37] Steinbach A., Mokkapati C., Singh P., Mukherjee S. (2026). Canonical and phosphoribosyl ubiquitination coordinate to stabilize a proteinaceous structure surrounding the *Legionella*-containing vacuole. eLife.

[38] Oide H., Kubori T., Nagai H., Nozawa T., Nakagawa I., Sasai M., Yamamoto M., Arasaki K. (2025). Phosphatidic acid production on the vacuole harboring *Legionella pneumophila* is a signal for recognition of interferon-induced GTPases. Proc. Natl. Acad. Sci. USA.

[39] Yek K.Q., Hodgson E.R., Ang C.S., Palmer C.S., Frazier A.E., Newton H.J., Stojanovski D. (2025). *Legionella* effector LpPIP recruits protein phosphatase 1 to the mitochondria to induce dephosphorylation of outer membrane proteins. PLoS Biol..

[40] Katic A., Vittori E.T., Halter S., Steiner K., Swart A.L., Radiom M., García-Rodríguez F.J., Jäggi T., Li X., A Mears J. (2026). The *Legionella* effector RidL binds the large fission GTPase Drp1 to promote mitochondrial fragmentation. EMBO Rep..

[41] Zhang R., Zhang M., Li X., Lu Z., Song C., Du Y., Chesterman C., van Staalduinen L., Wang Y., Pei H. (2025). A *Legionella pneumophila* T4SS effector protein (Ravl) triggers mitochondrial fragmentation through phosphoinositide phosphatase activity. Int. J. Biol. Macromol..

[42] Zhang Q., Kudryashova E., Sanford E., Wagner M.M., Minelli M.E., Smolka M.B., Kudryashov D.S., Mao Y. (2026). *Legionella* effector RavH is a tandem WH2-like domain-containing PI(3)P-dependent actin nucleator. Life Sci. Alliance.

[43] Liu J., Liu S., Shu R., Ma K., Lu Q., Ge J., Liu H., Fu J., Qiu J. (2026). *Legionella* effector protein SidG disrupts host cytoskeleton via targeting Arp2/3 complex. PLoS Pathog..

[44] Parducho K.R., Yang Z., Guinn E., Choi D., Nag S., Melia T.J., Roy C.R. (2026). *Legionella* Lem26 functions as an ATG8-activated effector that inhibits host autophagy. mBio.

[45] Choi M., Jeong M., Kang S., Jeon H., Shin D. (2024). *Legionella pneumophila* evades host-autophagic clearance using phosphoribosyl-polyubiquitin chains. Nat. Commun..

[46] Chen J., Li F., Wang S.Y., Chen T.T. (2026). Crystal structure of *Legionella pneumophila* glycosidase effector LegY. Acta Crystallogr. Sect. F Struct. Biol. Commun..

[47] Shin C.J., Price C.T.D., Da’as M.S.J., Abu Kwaik Y. (2026). Localization of the PelC and PelE effectors to the *Legionella*-containing vacuole through host-mediated prenylation and their role in intracellular proliferation. Infect. Immun..

[48] Gonçalves A.V., Margolis S.R., Quirino G.F.S., Mascarenhas D.P.A., Rauch I., Nichols R.D., Ansaldo E., Fontana M.F., Vance R.E., Zamboni D.S. (2019). Gasdermin-D and Caspase-7 are the key Caspase-1/8 substrates downstream of the NAIP5/NLRC4 inflammasome required for restriction of *Legionella pneumophila*. PLoS Pathog..

[49] Bass A.R., Egan M.S., Alexander-Floyd J., Lopes Fischer N., Doerner J., Shin S. (2023). Human GBP1 facilitates the rupture of the *Legionella*-containing vacuole and inflammasome activation. mBio.

[50] Hanford H.E., Price C.T.D., Uriarte S., Abu Kwaik Y. (2025). Inhibition and evasion of neutrophil microbicidal responses by *Legionella longbeachae*. mBio.

[51] Schmidt S., Mondino S., Gomez-Valero L., Escoll P., Mascarenhas D.P.A., Gonçalves A., Camara P.H.M., Rodriguez F.J.G., Rusniok C., Sachse M. (2024). The unique *Legionella longbeachae* capsule favors intracellular replication and immune evasion. PLoS Pathog..

[52] Kowalczyk B., Petzold M., Kaczyński Z., Szuster-Ciesielska A., Luchowski R., Gruszecki W.I., Fuchs B., Galuska C.E., Choma A., Tarasiuk J. (2023). Lipopolysaccharide of *Legionella pneumophila* serogroup 1 facilitates interaction with host cells. Int. J. Mol. Sci..

[53] Rehman S., Antonovic A.K., McIntire I.E., Zheng H., Cleaver L., Baczynska M., Adams C.O., Portlock T., Richardson K., Shaw R. (2024). The *Legionella* collagen-like protein employs a distinct binding mechanism for the recognition of host glycosaminoglycans. Nat. Commun..

[54] Vázquez Marrero V.R., Doerner J., Wodzanowski K.A., Zhang J., Lu A., Boyer F.D., Vargas I., Hossain S., Kammann K.B., Dresler M.V. (2025). Dendritic cells activate pyroptosis and effector-triggered apoptosis to restrict *Legionella* infection. mBio.

[55] Zhou P., She Y., Dong N., Li P., He H., Borio A., Wu Q., Lu S., Ding X., Cao Y. (2018). Alpha-kinase 1 is a cytosolic innate immune receptor for bacterial ADP-heptose. Nature.

[56] García-Weber D., Arrieumerlou C. (2021). ADP-heptose: A bacterial PAMP detected by the host sensor ALPK1. Cell. Mol. Life Sci..

[57] Liu M., Haenssler E., Uehara T., Losick V.P., Park J.T., Isberg R.R. (2012). The *Legionella pneumophila* EnhC protein interferes with immunostimulatory muramyl peptide production to evade innate immunity. Cell Host Microbe.

[58] Kowalczyk B., Chmiel E., Palusinska-Szysz M. (2021). The Role of Lipids in *Legionella*-Host Interaction. Int. J. Mol. Sci..

[59] Conover G.M., Martinez-Morales F., Heidtman M.I., Luo Z.Q., Tang M., Chen C., Geiger O., Isberg R.R. (2008). Phosphatidylcholine synthesis is required for optimal function of *Legionella pneumophila* virulence determinants. Cell. Microbiol..

[60] Flieger A., Gongab S., Faigle M., Mayer H.A., Kehrer U., Mussotter J., Bartmann P., Neumeister B. (2000). Phospholipase A secreted by *Legionella pneumophila* destroys alveolar surfactant phospholipids. FEMS Microbiol. Lett..

[61] Remen T., Mathieu L., Hautemaniere A., Deloge-Abarkan M., Hartemann P., Zmirou-Navier D. (2011). Pontiac fever among retirement home nurses associated with airborne *legionella*. J. Hosp. Infect..

[62] Gorman D., Green A., Puri N., Dellinger P. (2022). Severe ARDS secondary to *Legionella* pneumonia requiring VV ECMO in the setting of newly diagnosed hairy cell leukemia. J. Investig. Med. High Impact Case Rep..

[63] Qi Z., Caiping Z., Wei W. (2026). Rhabdomyolysis caused by severe *Legionella* pneumonia combined with influenza A. SAGE Open Med. Case Rep..

[64] Pulsipher A.M., Khattar G., VanDolah H., Gotway M., Ham K., Alqalyoobi S., Cartin-Ceba R., Patel R., Simner P.J., Sen A. (2026). *Legionella* Pneumonia in the Modern Era: Clinical Features and Predictors of Mortality. Clin. Infect. Dis..

[65] Falcone M., Russo A., Tiseo G., Cesaretti M., Guarracino F., Menichetti F. (2021). Predictors of intensive care unit admission in patients with *Legionella* pneumonia: Role of the time to appropriate antibiotic therapy. Infection.

[66] Yamasue M., Komiya K., Kinjo T., Ito A., Yamaguchi T., Iwanaga N., Ishii M., Tateda K., Kawakami K. (2023). Rebound mortality rate of *Legionella* pneumonia in Japan. Respir. Investig..

[67] Song Y., Wang H., Lin L., Cheng Y., Shen Y. (2026). Clinical characteristics and outcomes of severe *Legionella pneumophila* pneumonia diagnosed by metagenomic next-generation sequencing in children: A case series of 8 patients. Front. Cell. Infect. Microbiol..

[68] Angert D.M., Dowdell K.S., Nguyen L., Park C., Brown J., Russell C., Bautista M., Hegarty B., Cambronne E.D., Kirisits M.J. (2025). Particle-associated and free-living *Legionella* spp. in drinking water. Water Res..

[69] Stiboy E., Klingstrom M., Chee K., Ferson M., Chaverot S., Huang B., Hannan T., Pershen P., Hope K., Baldwin Z. (2025). Where the cooling water drifts: An outbreak of *Legionella pneumophila* serogroup 1 in the central business district of Sydney, December 2023–January 2024. Commun. Dis. Intell..

[70] Biham O., Azulay H., Nativ R., Ischa Abar V., Borer A., Nesher L., Karp G. (2026). *Legionella* on tap: Nosocomial pneumonia cluster linked to a hospital cold water dispenser in hematology patients. Infect. Control Hosp. Epidemiol..

[71] Slekovec C., Biguenet A., Jeanvoine A., Hocquet D., Bertrand X. (2026). Case of *Legionella pneumophila* serogroup 1 infection linked to water flosser, France. Emerg. Infect. Dis..

[72] Athanasiou M., Palaiologou A., Chatziprodromidou I., Velissari J., Vantarakis A. (2026). Determinants of *Legionella* spp. presence in hospital water surveillance: A five-year, three-centre study from Greece. J. Hosp. Infect..

[73] Arqué E., Ragull S., Villodres-Moreno D., Alsina-Samaniego A., Satorra P., Tebé C., Rosell-Duran G., Pérez-Lallave D., Pedro-Botet L., Sabrià M. (2026). Impact of meteorological factors on *Legionella* colonisation of water systems and the incidence of Legionnaires’ disease. Water Res..

[74] Ogawa H., Takeda K., Yoneoka R., Shikano K., Abe M., Kasai H., Suzuki T. (2024). Severe Bacterial Pneumonia Due to *Legionella longbeachae* Transmitted from Potting Soil in Japan. Intern. Med..

[75] Crull S., Hammer E., Mann A.E., O’Connell L.M., Soule A., Griffith E., Blouin T., Brigmon R.L., Richards V.P. (2025). Seasonal host shifts for *Legionella* within an industrial water-cooling system. Environ. Microbiol. Rep..

[76] European Centre for Disease Prevention and Control (2025). Legionnaires’ Disease. Annual Epidemiological Report for 2023.

[77] Graham F.F., Harte D., Zhang J., Fyfe C., Baker M.G. (2023). Increased Incidence of Legionellosis after Improved Diagnostic Methods, New Zealand, 2000–2020. Emerg. Infect. Dis..

[78] Chambers S.T., Slow S., Scott-Thomas A., Murdoch D.R. (2021). Legionellosis Caused by Non-*Legionella pneumophila* Species, with a Focus on *Legionella longbeachae*. Microorganisms.

[79] Arrigo I., Serra N., Mariyam L., Sarvandani M.M., Tricoli M.R., Palermo R., Diquattro O., Gallina G., Di Carlo P., Firenze A. (2025). Comparison between Legiolert and real time PCR in the detection of *Legionella pneumophila* from environmental water samples. PLoS ONE.

[80] Petzold M., Zacharias N., Uhle S., Kieper L., Mutters N.T., Kistemann T., Schreiber C. (2025). PCR-based *Legionella* risk evaluation of drinking water systems: An empirical field evaluation. Microorganisms.

[81] Meegoda C.S., Waak M.B., Kim T., Hozalski R.M., Hallé C. (2026). Flushing as a Control Measure for *Legionella* spp.: Impacts of Water Age, Chloramine Disinfection, and Elevated Temperature. Environ. Sci. Technol..

[82] Clark N., McCarthy L.H., Liss S.N. (2026). Alternative disinfection in hot water networks: Persistence and antimicrobial efficacy of silver-stabilized hydrogen peroxide for *Legionella pneumophila* control. ACS ES&T Water.

[83] Seah J., Lai D.C.M., Toh H.X., Soh C.Y., Amin I.M., Ling M.L. (2026). Targeted blower-assisted thermal disinfection for persistent distal outlet *Legionella* contamination in a tertiary hospital water system. Infect. Control Hosp. Epidemiol..

[84] Wolf A., Abdullah T.E.A., Moritz A., Georgopoulos A. (2026). Long-term control of *Legionella pneumophila* and *Pseudomonas aeruginosa* in dental unit waterlines following optimization of a hypochlorous acid-based water safety program. Front. Oral Health.

[85] Spagnuolo V., Trerotoli P., Diella G., Fasano F., Lorusso L., Savino A.F., Triggiano F., Montagna M.T., De Giglio O. (2026). Quantitative microbial risk assessment and cost-benefit analysis of *Legionella* contamination in the water system of a large hospital in Southern Italy. Int. J. Hyg. Environ. Health.

[86] You T., Parmar P., Decke G., Baikie K., Ryckewaert L., Marette A., Niculita-Hirzel H. (2026). Water-vapor treatment of shower systems in healthcare facilities: Reshaping built-environment microbiomes to improve *Legionella* control and protect at-risk patients. Microbiome.

[87] Feeley J.C., Gibson R.J., Gorman G.W., Langford N.C., Rasheed J.K., Mackel D.C., Baine W.B. (1979). Charcoal-yeast extract agar: Primary isolation medium for *Legionella pneumophila*. J. Clin. Microbiol..

[88] Scaturro M., Girolamo A., Bella A., Rota M.C., Marella A.M., Amari R., Barberis D., Romano C., Borney F., Damasco F. (2025). Interlaboratory comparison of culture- and PCR-based methods for *Legionella pneumophila* detection in drinking water samples. Appl. Environ. Microbiol..

[89] Kawasaki T., Nakagawa N., Murata M., Yasuo S., Yoshida T., Ando K., Okamori S., Okada Y. (2022). Diagnostic accuracy of urinary antigen tests for legionellosis: A systematic review and meta-analysis. Respir. Investig..

[90] Murakami Y., Morosawa M., Nozaki Y., Takesue Y. (2024). *Legionella* pneumonia undetected by repeated urinary antigen testing with Ribotest *Legionella*. Cureus.

[91] Foglia Manzillo L., Teggia Droghi M., Iannuzzi F., Castelli D., Foti G., Bonfanti P., Giani M. (2026). Urinary antigen-negative *Legionella* pneumonia in critically ill patients: Value of systematic molecular testing for optimal antimicrobial therapy. J. Antimicrob. Chemother..

[92] Avni T., Bieber A., Green H., Steinmetz T., Leibovici L., Paul M. (2016). Diagnostic Accuracy of PCR Alone and Compared to Urinary Antigen Testing for Detection of *Legionella* spp.: A Systematic Review. J. Clin. Microbiol..

[93] Pan Y., Xing Y., Lai Y., Dong H., Sheng H., Xu W. (2024). Metagenomic next-generation sequencing reveals co-infection with *Legionella pneumophila* and *Fusobacterium necrophorum* in a patient with severe pneumonia: A case report. BMC Pulm. Med..

[94] Bu C., Lei S., Chen L., Xie Y., Zheng G., Hua L. (2023). Case report: Fatal *Legionella* infection diagnosed by metagenomic next-generation sequencing in a patient with chronic myeloid leukemia. Front. Med..

[95] Zhang P., Liu B., Zhang S., Chang X., Zhang L., Gu D., Zheng X., Chen J., Xiao S., Wu Z. (2024). Clinical application of targeted next-generation sequencing in severe pneumonia: A retrospective review. Crit. Care.

[96] Li N.N., Mo X.G., Liao Z., Li A.J., Mao L., Li J., Li B., Yin Y., Yao Q. (2026). Severe *Legionella pneumophila* pneumonia identified by targeted next-generation sequencing after nondiagnostic conventional testing: A case report. IDCases.

[97] Martins M.F., Govindan R., Almaghlouth N.K., Kirby J.E., Kentoffio K.J., Farmakiotis D., Le-Mahajan A. (2026). A fatal case of *Legionella micdadei* prosthetic valve endocarditis diagnosed by plasma microbial cell-free DNA metagenomic sequencing. Lancet Infect. Dis..

[98] Li J., Wang X., Wang X., Qu H., Gao L., Zhao Z., Luo P., Zheng Y. (2025). A rapid and sensitive CRISPR-Cas12a for the detection of *Legionella pneumophila*. Pol. J. Microbiol..

[99] Pulsipher A.M., Khattar G., Harris E., White V., Stout C., Vikram H.R., Patel R., Simner P.J. (2026). *Legionella* 5S rRNA PCR melting temperature analysis discriminates high-risk species associated with disease severity. J. Clin. Microbiol..

[100] Fu K.P., Neu H.C. (1979). Inactivation of beta-lactam antibiotics by *Legionella pneumophila*. Antimicrob. Agents Chemother..

[101] Garcia-Vidal C., Sanchez-Rodriguez I., Simonetti A.F., Burgos J., Viasus D., Martin M.T., Falco V., Carratalà J. (2017). Levofloxacin versus azithromycin for treating *legionella* pneumonia: A propensity score analysis. Clin. Microbiol. Infect..

[102] Gershengorn H.B., Keene A., Dzierba A.L., Wunsch H. (2015). The association of antibiotic treatment regimen and hospital mortality in patients hospitalized with *Legionella* pneumonia. Clin. Infect. Dis..

[103] Nagel J.L., Rarus R.E., Crowley A.W., Alaniz C. (2014). Retrospective analysis of azithromycin versus fluoroquinolones for the treatment of *legionella* pneumonia. P T.

[104] Rello J., Gattarello S., Souto J., Sole-Violan J., Valles J., Peredo R., Zaragoza R., Vidaur L., Parra A., Roig J. (2013). Community-acquired *Legionella* pneumonia in the intensive care unit: Impact on survival of combined antibiotic therapy. Med. Intensiv..

[105] Bouteiller M., Cossé C., Beduneau G., Giry M., Caillard C., Barrau D., Malherbe J., Nseir S., Vodovar D., Tamion F. (2026). Monotherapy versus combination therapy in severe *Legionella* pneumonia: A multicenter retrospective observational study. Crit. Care.

[106] Angus D.C., REMAP-CAP Investigators (2025). Effect of hydrocortisone on mortality in patients with severe community-acquired pneumonia: The REMAP-CAP corticosteroid domain randomized clinical trial. Intensive Care Med..

[107] Dequin P.F., Meziani F., Quenot J.P., Kamel T., Ricard J.D., Badie J., Reignier J., Heming N., Plantefève G., Souweine B. (2023). Hydrocortisone in severe community-acquired pneumonia. N. Engl. J. Med..

[108] Cortès-Tarragó R., Arqué E., Vinyeta L., Basart Altieri M., Elizondo S.M., Sopena N., Reynaga E., Ríos J.D.d.L., Quero S., Párraga-Niño N. (2026). In vitro characterization of delafloxacin activity against intracellular *Legionella*: Exploring new treatment options for Legionnaires’ disease. Int. J. Infect. Dis..

[109] Lv J., Liu C., Fan L., Luo P., Liu S., Wu C. (2024). Omadacycline for the treatment of severe *Legionella pneumophila* pneumonia complicated with multiple organ dysfunction: A case report. Diagn. Microbiol. Infect. Dis..

[110] Li B., Liu L., Li F., He C., Chen Y., Tian X., Liu Z., Zhang W. (2025). Omadacycline successfully treated severe *Legionella* pneumonia after moxifloxacin treatment failure: Case series. Front. Pharmacol..

[111] Jia X., Ren H., Nie X., Li Y., Li J., Qin T. (2019). Antibiotic resistance and azithromycin resistance mechanism of *Legionella pneumophila* serogroup 1 in China. Antimicrob. Agents Chemother..

[112] Minetti C., Barton R., Farley C., Spiller O.B., Rodrigues R., Gonçalves P. (2024). Antimicrobial susceptibility testing reveals reduced susceptibility to azithromycin and other antibiotics in *Legionella pneumophila* serogroup 1 isolates from Portugal. Eur. J. Clin. Microbiol. Infect. Dis..

[113] Portal E., Descours G., Ginevra C., Mentasti M., Afshar B., Chand M., Day J., Echahidi F., Franzin L., Gaia V. (2021). *Legionella* antibiotic susceptibility testing: Is it time for international standardization and evidence-based guidance? *J*. Antimicrob. Chemother..

[114] Lorentzen M.H., Rosenvinge F.S., Lassen A.T., Graumann O., Laursen C.B., Mogensen C.B., Skjøt-Arkil H. (2023). Empirical antibiotic treatment for community-acquired pneumonia and accuracy for *Legionella pneumophila*, *Mycoplasma pneumoniae*, and *Chlamydophila pneumoniae*: A descriptive cross-sectional study of adult patients in the emergency department. BMC Infect. Dis..

[115] Shi L., Zhang D., Yang Q., Yang J., Zhu H. (2024). Distinction of clinical features and microbiological methods between *Chlamydia psittaci* and *Legionella pneumophila* pneumonia confirmed by metagenomic next-generation sequencing. Ann. Med..

[116] Tanaka S., Oide H., Ikeda S., Tagaya M., Nagai H., Kubori T., Arasaki K. (2025). Subversion of the host endocytic pathway by *Legionella pneumophila*-mediated ubiquitination of Rab5. J. Cell Biol..

[117] Stegmann F., Diersing C., Lepenies B. (2024). *Legionella pneumophila* modulates macrophage functions through epigenetic reprogramming via the C-type lectin receptor Mincle. iScience.

[118] Cameron G., Faucher S.P. (2025). Copper resistance in *Legionella pneumophila*: Role of genetic factors and host cells. Sci. Total Environ..

[119] Madagi L., Azrad M., Karp M., Peretz A., Sharaby Y. (2026). Longitudinal Antimicrobial Susceptibility Shifts in Environmental *Legionella pneumophila* from a Drinking Water Distribution System. Pathogens.

